# *CHD7* Disorder—Not CHARGE Syndrome—Presenting as Isolated Cochleovestibular Dysfunction

**DOI:** 10.3390/genes15050643

**Published:** 2024-05-19

**Authors:** Jef Driesen, Helen Van Hoecke, Leen Maes, Sandra Janssens, Frederic Acke, Els De Leenheer

**Affiliations:** 1 Department of Head and Skin, Faculty of Medicine and Health Sciences, Ghent University Hospital, 9000 Ghent, Belgium; 2 Department of Rehabilitation Sciences, Faculty of Medicine and Health Sciences, Ghent University Hospital, 9000 Ghent, Belgium; 3 Department of Biomolecular Medicine, Faculty of Medicine and Health Sciences, Ghent University Hospital, 9000 Ghent, Belgium

**Keywords:** CHARGE syndrome, *CHD7* Genotype–phenotype, cochleovestibular audiometry, nonsyndromic hearing loss

## Abstract

CHARGE syndrome, characterized by a distinct set of clinical features, has been linked primarily to mutations in the *CHD7* gene. Initially defined by specific clinical criteria, including coloboma, heart defects, choanal atresia, delayed growth, and ear anomalies, CHARGE syndrome’s diagnostic spectrum has broadened since the identification of *CHD7*. Variants in this gene exhibit considerable phenotypic variability, leading to the adoption of the term “*CHD7* disorder” to encompass a wider range of associated symptoms. Recent research has identified *CHD7* variants in individuals with isolated features such as autism spectrum disorder or gonadotropin-releasing hormone deficiency. In this study, we present three cases from two different families exhibiting audiovestibular impairment as the primary manifestation of a *CHD7* variant. We discuss the expanding phenotypic variability observed in *CHD7*-related disorders, highlighting the importance of considering *CHD7* in nonsyndromic hearing loss cases, especially when accompanied by inner ear malformations on MRI. Additionally, we underscore the necessity of genetic counseling and comprehensive clinical evaluation for individuals with *CHD7* variants to ensure appropriate management of associated health concerns.

## 1. Introduction

CHARGE syndrome is an autosomal dominant genetic condition characterized by a nonrandom association of clinical features. In the premolecular era, the acronym CHARGE was proposed for the combination of coloboma, heart defect, choanal atresia, delayed growth and development, genital hypoplasia, and ear anomalies of unknown cause [1]. Over the years, clinical diagnostic criteria were refined and classified for what later became known as the CHARGE association [2,3].

In 2004, *CHD7* was identified as a gene responsible for CHARGE syndrome [4], with pathogenic *CHD7* variants being identified in 70–90% of suspected cases [5,6]. However, when strict clinical diagnostic criteria are met, a pathogenic *CHD7* variant is present in over 90% of cases [7]. *CHD7* encodes a member of the Chromodomain Helicase DNA binding [CHD] protein family, an ATP-dependent nucleosome remodeling factor whose members are involved in tissue-specific regulation of gene expression during development [8]. As a nucleosome remodeling factor, it utilizes the energy released from ATP hydrolysis to regulate the position and density of nucleosomes at target loci [9]. A recent study revealed that, in addition to its nucleosome remodeling activity, *CHD7* can also directly recruit histone methyltransferase activity to its targets [10].

Following this discovery, the phenotypic spectrum expanded to phenotypes that do not fulfill the previously proposed CHARGE syndrome clinical diagnostic criteria, with heterozygous [likely] pathogenic variants in *CHD7* seeming to cause a wide spectrum of phenotypes, including CHARGE syndrome. This variability is observed even among individuals in the same family and among individuals from different families with the same *CHD7* variant [11]. Therefore, the term ‘*CHD7* disorder’ has been adopted more recently, referring to the entire phenotypic spectrum that can be associated with heterozygous *CHD7* pathogenic variants and is not equivalent to a diagnosis of CHARGE syndrome [12,13].

An estimate on *CHD7* mutations suggested a birth incidence of 1 in 18,400. For CHARGE syndrome, however, individuals who had not undergone *CHD7* analysis due to mild symptoms and those with typical CHARGE syndrome but lacking a *CHD7* mutation need to be accounted for. Factoring in these considerations, the estimated incidence of CHARGE syndrome ranges from 1 in 15,000–17,000 live births [14]

*CHD7* variants have already been reported in individuals with isolated phenotypical features, including autism spectrum disorder [15,16] or gonadotropin-releasing hormone deficiency [17]. Here, we present two cases with a class 3 and one case with a class 4 *CHD7* variant, and an atypical presentation confined to audiovestibular impairment. We also review the literature on previously reported atypical features and the possible role of *CHD7* in the development and pathogenesis of these phenotypes.

## 2. Materials and Methods

We ascertained three cases of *CHD7* disorder with isolated audiovestibular phenotype at the ENT department of Ghent University Hospital [Belgium]. Genetic testing was performed at Antwerp University Hospital, Belgium. Exome sequencing was performed using the ‘sequencing by synthesis’ technology (Illumina, sequencing subcontracted at Radboudumc Nijmegen) after in-house enrichment of the exome with the Twist man Core Exome kit, which includes additional probes for human RefSeq transcripts and the mitochondrial genome (Twist Bioscience). Variants were detected through analysis of a set of 146 genes known to cause hearing loss (WESHL panel v2.0) and additional genome-wide filtering based on HPO (Human Phenotype Ontology) (MOON software, Diploid/Invitae). The classification of detected sequence variants is performed according to the 5-class system: non-pathogenic (class 1, polymorphism), likely non-pathogenic (class 2), clinical significance unclear (class 3), likely pathogenic (class 4), and pathogenic (class 5, mutation). The position of the displayed variants is based on NCBI build GRCh37.

MRI of the inner ear with sagittal MPRAGE, axial T2 fatsat, and FLAIR techniques was performed. High-resolution 3D T2 imaging through the inner ear regions bilaterally was acquired.

To further assess the prevalence and variability of hearing and vestibular anomalies associated with *CHD7* variants, we reviewed the literature on individuals with *CHD7* variants. This work was approved by the Ethics committee of Ghent University Hospital, reference number CR-2023-0002. 

## 3. Cases

### 3.1. Case 1

A 5-year-old boy was referred to the Ghent ENT department because of unilateral left-sided hearing loss. He was delivered at 40 weeks and 5 days of gestation after an uncomplicated pregnancy. At one month of age, he was referred to another center because of failed newborn hearing screening [automated auditory brainstem responses [ABR] at 35dBnHL]. The subsequent ABR indicated bilateral normal hearing thresholds at 10dBnHL; however, no report was available. Speech development was normal, but he was previously identified with a minor motor delay and hypotonia, being able to crawl at 12 months and walk at the age of 22 months. Physiotherapy was initiated at the age of 2.5 years, and his walking pattern has now normalized. No reports of vertigo, nausea, or vomiting have been documented. There was no history of otitis media or previous ear surgery, nor a family history of syndromic disease or congenital/early onset hearing loss. 

**Figure 1:**
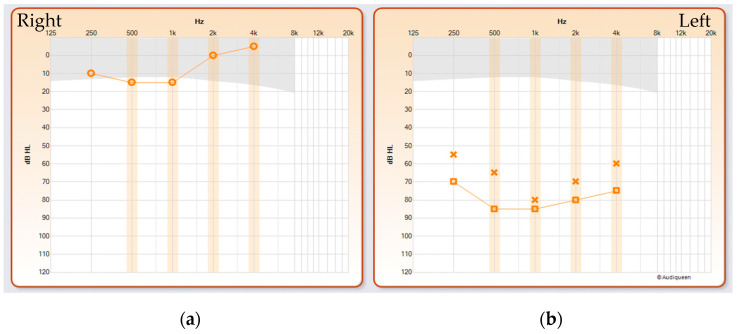
Audiometry demonstrating normal hearing on the right side (**a**) and unilateral sensorineural hearing loss on the left side (**b**). Squares indicate masked air-conduction thresholds, circles (right ear) and crosses (left ear) indicate unmasked air-conduction thresholds. The vertical axis shows the degree of hearing loss in decibels [dB], and the horizontal axis shows the frequency range.

Clinical ENT examination at the age of 5 years showed normal findings. General inspection revealed a slight ptosis of the left eyelid that had been present since birth. Pure tone audiometry disclosed a normal hearing threshold of 12 dB HL pure tone average [PTA] on the right side and a unilateral profound hearing loss of 85 dB HL PTA on the left side (Figure 1). Tympanometry was bilaterally normal, while transient otoacoustic emissions were absent on the left side.

Vestibular examination revealed a severe bilateral vestibular dysfunction [slightly better on the right than the left] with only preserved function of the anterior semicircular canal on both sides and no, to limited, residual function in all other parts [i.e., horizontal and posterior SCC, saccule and utricle]. Vestibular testing included caloric and rotatory testing [for low and mid frequency characterization of the horizontal SCC, video head impulse [vHIT] testing, for high frequency investigation of the horizontal as well as vertical canals, and cervical [c] and ocular [o] vestibular evoked myogenic potentials [VEMP] for examination of saccular and utricular function, respectively. 

Etiologically, cytomegalovirus [CMV] PCR on the dried blood spot yielded negative results. Molecular analysis, using a whole exome sequencing panel [WESHL panel v2.0] containing 146 deafness genes, identified a heterozygous *CHD7* variant [NM_017780.4]: c.8077-2A>T. This substitution alters the splice acceptor site of the last exon of *CHD7* and is considered likely pathogenic. Both parents tested negative, so it is considered a de novo variant.

**Figure 2:**
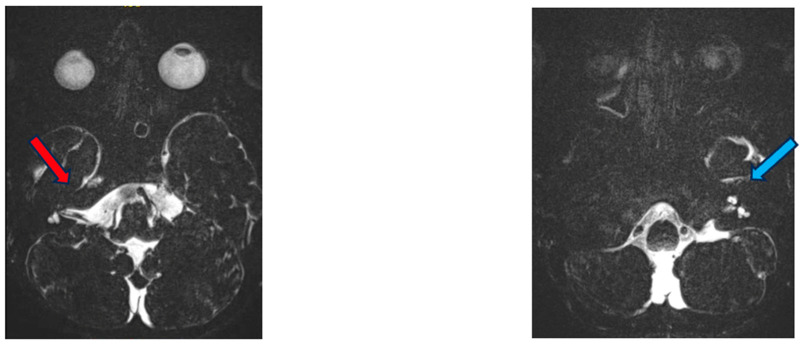
MRI of the inner ear for case 1, T2 tse3d weighted image, dysplasia of the lateral and vestibulum bilaterally (blue arrow depicts dysplastic canal on the left, red on the right).

MRI revealed dysplasia of the lateral and posterior semicircular canals and the vestibulum bilaterally, with a normally constructed superior semicircular canal on the right and a smaller superior semicircular canal on the left. No dysplasia of the cochlea or intracranial abnormalities were evident (Figure 2). 

Subsequently, other congenital abnormalities associated with CHARGE syndrome were ruled out. Ophthalmologic examination revealed normal vision. Echocardiography revealed a structurally normal heart, with normal electrocardiogram results. Cognitive development was normal. Ultrasound of both kidneys showed normal morphology without evidence of hydronephrosis. Endocrinological examination revealed no evidence of thyroid disease, calcium/phosphorus disorders, or genital anomalies. Due to his prepubertal status, the gonadotropic axis cannot be estimated yet.

### 3.2. Case 2

**Figure 3:**
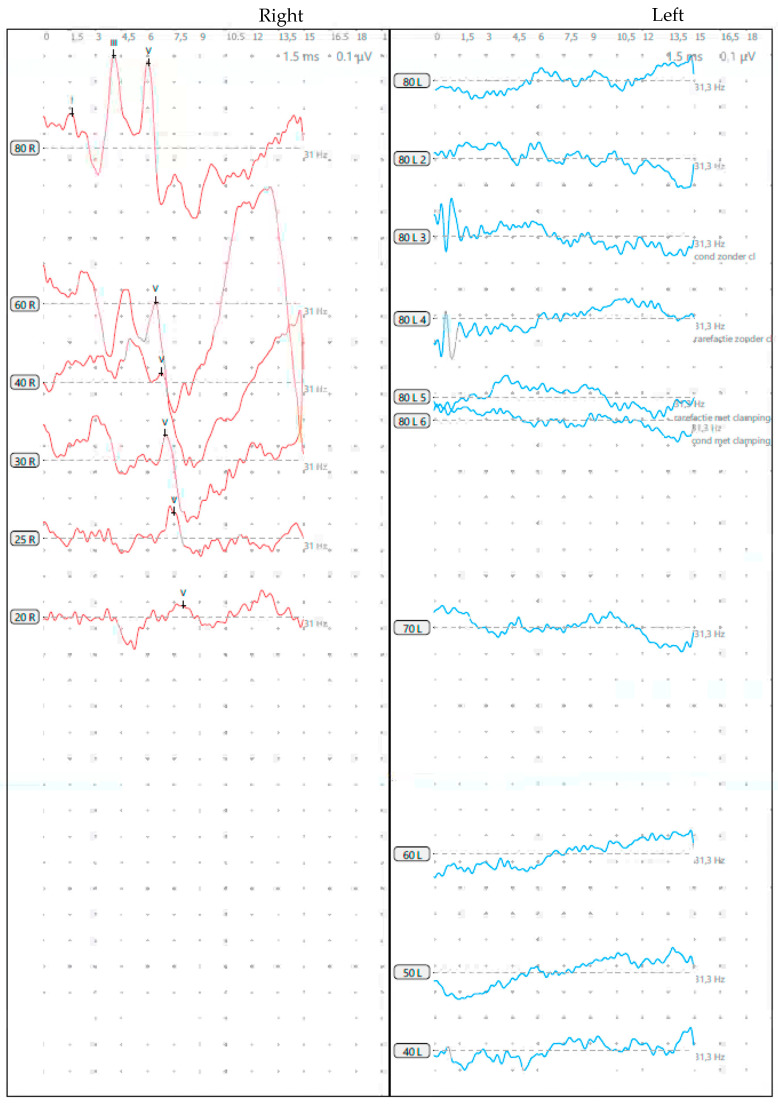
ABR with a normal hearing threshold at 20 dB nHL is shown in red on the right panel; absent thresholds at 80 dB nHL are depicted in blue on the left, with no detectable I–V complex.

A boy presented shortly after birth with congenital left-sided hearing loss. He was delivered at 36 weeks of gestation in an otherwise uncomplicated pregnancy. Clinical ENT examination was normal, and no dysmorphic features were observed. ABR following a referral from neonatal hearing screening revealed an absent threshold at 80dBnHL on the left and a normal hearing threshold of 25dBnHL on the right (Figure 3). Otoacoustic emissions were present on the right but absent on the left side. Vestibular examination showed bilaterally normal function of the anterior semicircular canals and bilaterally significantly reduced function of the posterior and horizontal semicircular canals, evaluated by video head impulse test [vHIT]. Cervical Vestibular Evoked Myogenic Potential [cVEMP] and ocular Vestibular Evoked Myogenic Potential [oVEMP] showed bilateral reproducible responses with symmetrical and normal amplitude.

Etiologically, CMV PCR yielded negative results. Molecular analysis by means of a deafness gene panel [WESHL panel v2.0] identified a heterozygous variant in the *CHD7* [NM_017780.4]: c.6193C>T p.[Arg2065Cys] and the myosin IIIA [*MYO3A*] [NM_017433.5]: c.3133G>T p.[Val1045Leu] gene, both classified as class 3 variants or variants of unknown significance. Family genetic testing showed no *CHD7* variant in his older, healthy sister and his healthy mother, whereas the variant was present in his father, who was apparently asymptomatic but was later proven affected [case 3]. 

**Figure 4:**
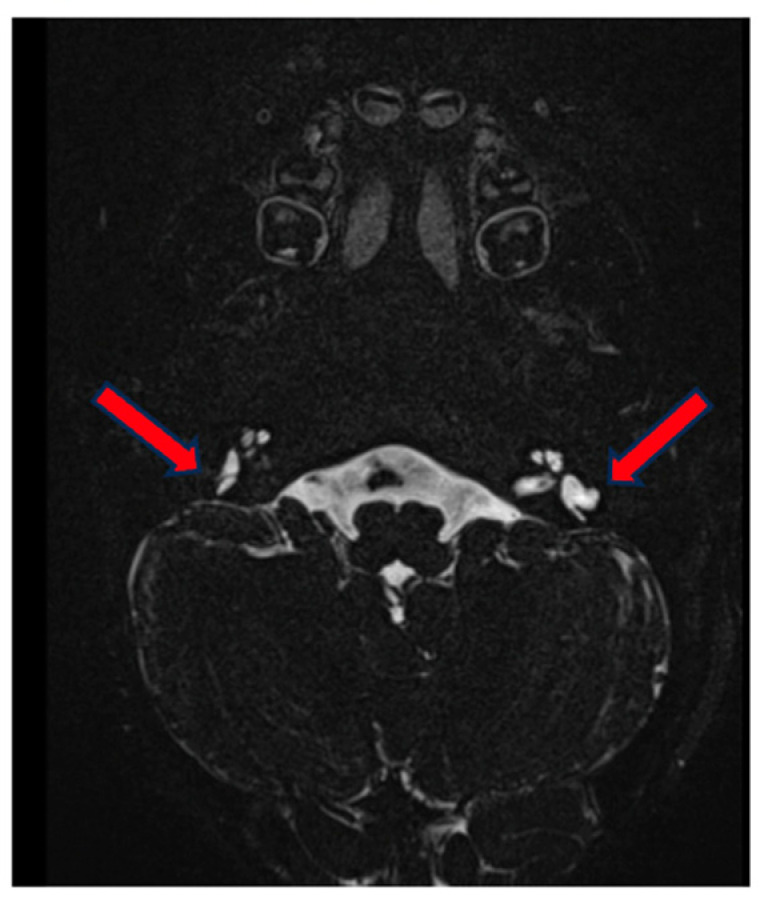
MRI of the inner ear for case 2, T2 tse3d weighted image, dysplasia of lateral semicircular canals (red arrows).

**Figure 5:**
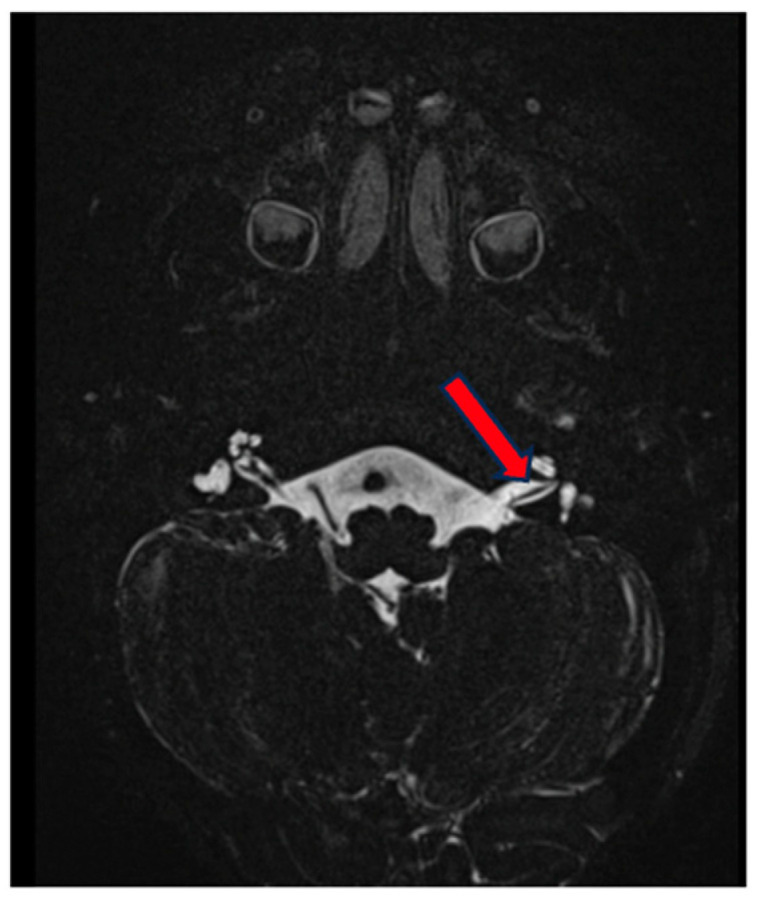
MRI of the inner ear for case 2, T2 tse3d weighted image, hypoplasia of the left cochlear nerve (red arrow).

MRI correlated to the findings of the vestibular exam, revealing a normal fluid signal in the cochlea and vestibulum, as well as in the upper semicircular canal. Aberrant morphology with a blunt appearance of the lateral semicircular canal, which lacks a central opening, and partly also of the posterior semicircular canal, which appears smaller, with abnormal morphology and loss of the circular aspect. No widening of the vestibular aqueduct (Figure 4). There is a suspicion of hypoplasia of the left cochlear nerve, whereas the cochlea appears normally developed (Figure 5).

Ophthalmological examination showed no manifest abnormalities, aside from an impression of a slightly smaller eye slit. Endocrinological assessment revealed a boy in good general condition, with normal height and weight. The genitals were normally developed, and the testes were scrotal. On cardiac examination, a limited Ebstein malformation was initially noted, which was self-limiting with recent normal cardiac follow-up. Nephrological evaluation revealed no particularities, with a normal aspect of both kidneys on ultrasonography.

### 3.3. Case 3

**Figure 6:**
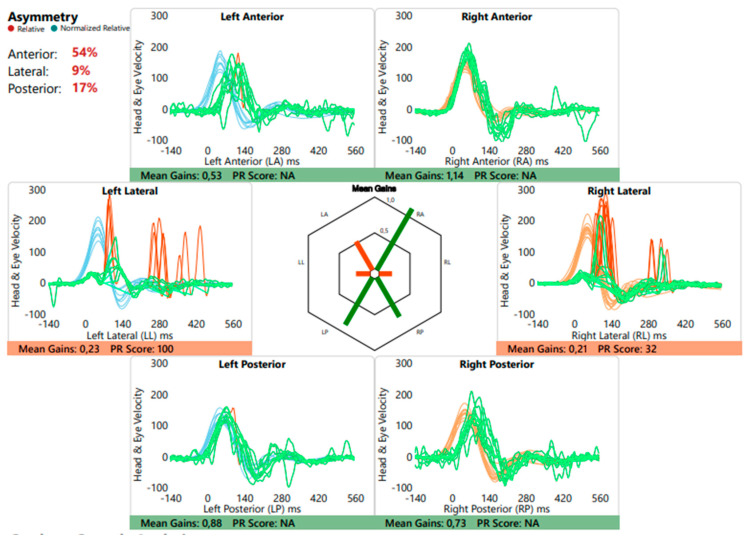
Video Head Impulse Test (vHIT) of the three left and right semicircular canals. The blue and orange lines indicate head movement on the left and right side of the head, respectively, in relation to its accompanied eye movement (green lines) as part of the vestibulo–ocular reflex. Results are quantified as ‘mean gains’. A perfect VOR gain is a gain of 1.0 (eye movement is exactly equal and opposite to head movements), while a VOR gain lower than 0.80 has been proposed as the cut-point between normal and abnormally low VOR gain. The vHIT from case 3 demonstrates only preserved function of the right anterior and both posterior semicircular canals.

Our third case is the father of case 2, an asymptomatic 31-year-old man with subjectively normal hearing, balance, and vision. Familial genetic investigation revealed similar class 3 heterozygous variants in the *CHD7* [NM_017780.4]: c.6193C>T p.[Arg2065Cys] and *MYO3A* [NM_017433.5]: c.3133G>T p.[Val1045Leu] genes. Audiometric testing was normal. Vestibular examination showed a bilateral significantly reduced gain with corrective saccades of the lateral semicircular canals, abnormal function of the left anterior canal, and normal function of the right anterior and both posterior canals (Figure 6). cVEMP and oVEMP showed bilateral reproducible responses with symmetrical and normal amplitude.

On MRI, we observed a correlating image with a normal aspect of the cochlea and vestibulum. On the right side, there is a normally formed superior semicircular canal but hypoplasia of the lateral semicircular canal, which is small and blunt, without a bony island. The posterior semicircular canal is small but forms a complete ring.

**Figure 7:**
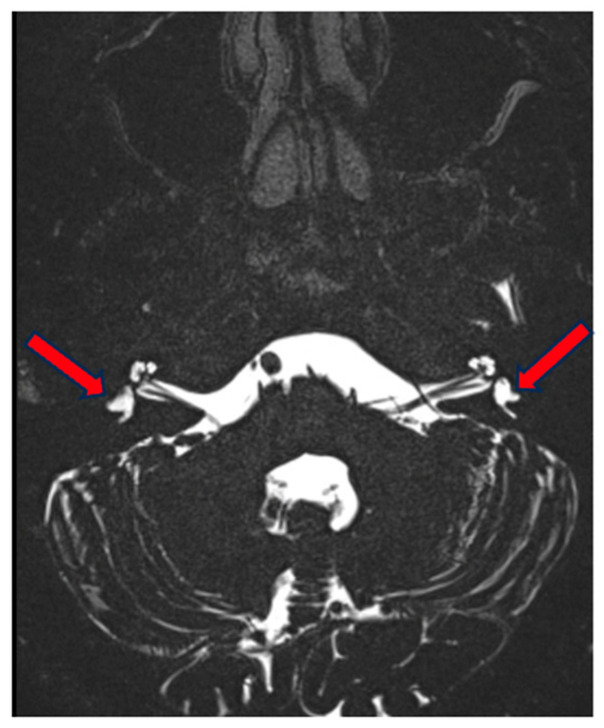
MRI of the inner ear for case 3, T2 tse3d weighted image, hypo-/dysplastic lateral semicircular canals bilaterally (red arrows).

On the left side, the superior semicircular canal is only partially formed [only a posterior part] and not a complete ring. The lateral semicircular canal is hypoplastic, only partially formed posteriorly and blunt. The posterior semicircular canal is also relatively small [larger than on the right] but forms a complete ring (Figure 7). There was also an incidental finding of hypoplasia of the right facial nerve, which was radiologically not demonstrable in the internal auditory canal or internal auditory meatus, without clinical correlation.

Following a motor vehicle trauma with kidney injury in the past, urological examination and imaging were performed with otherwise normal findings. There is a cardiac history of ablations in the context of atrioventricular re-entry tachycardia associated with Wolff–Parkinson–White syndrome. 

## 4. Discussion

The spectrum of clinical features associated with CHARGE syndrome has broadened since its initial description in 1981 [1]. Updates to clinical diagnostic criteria have enhanced our understanding of both major and minor features linked to CHARGE syndrome, showcasing the considerable variability in phenotypic severity reported since the identification of *CHD7*. The use of typical vs. atypical and major vs. minor criteria for a clinical diagnosis also reflects the complexity of commonly observed phenotypes [3]. Furthermore, with the increasing availability of molecular testing, individuals with pathogenic *CHD7* variants who do not meet the clinical criteria and those with less typical presentations are recognized. The term “*CHD7* disorder” has been adopted more recently to better account for this spectrum [12,13] and may be appropriate for individuals with pathogenic *CHD7* variants and subsets of CHARGE features. 

Atypical presentations in patients with a *CHD7* variant have been reported before [7,12], even without or with only one major diagnostic criterion for CHARGE syndrome, according to Verloes (Table 1) [3]. Some patients are only diagnosed based on more severely affected offspring [7]. Obviously, inheritance patterns in families are often related to a partial CHARGE phenotype and, thus, less severe symptomatology. In these families, large phenotypical variability is apparent [11,18]. For example, symptoms in one family ranged from unilateral hearing loss in one individual to bilateral cleft lip/palate, bilateral coloboma, growth deficiency, and external ear anomaly in a related individual [18]. The patients described here could be considered non-syndromic as they only exhibited cochleovestibular symptoms. Consequently, *CHD7* should also be considered in patients presenting with cochleovestibular symptoms only, especially when typical vestibular anomalies on MRI are present. 

Focusing on ENT-related symptoms and signs in patients with a *CHD7* pathogenic/likely pathogenic variant, the most prevalent include inner ear anomalies, external ear anomalies, hearing loss, cranial nerve dysfunction, and developmental delay [6,12,13]. Typical findings include a short auricle and wide ear with little or no lobe, a snipped-off helix, a prominent antihelix often not continuous with the tragus, a triangular concha, and reduced cartilage surface. The ears typically protrude and tend to be asymmetric. There is no characteristic hearing loss in CHARGE syndrome. The most prevalent features include asymmetrical mixed losses of severe-to-profound degree. These losses often entail conductive components resulting from a combination of ossicular anomalies and middle ear effusion, which tend to be asymmetrical and fluctuating. Additionally, sensorineural hearing loss, often stemming from cochlear malformations, typically manifests as greater impairment in the high frequencies. Temporal bone abnormalities include cochlear hypoplasia and the absence or underdevelopment of semicircular canals. Only recently, *CHD7* variant alleles have also been implicated in the etiology of enlarged vestibular aqueduct [EVA] and associated sensorineural hearing loss [19]. The authors advised evaluating potential CHARGE manifestations in individuals with EVA, especially in cases lacking SLC26A4 pathogenic variants.

Obviously, even in *CHD7* patients presenting as nonsyndromic, a complete work-up should be performed to demonstrate or exclude other manifestations. The cardiac findings in case 3 have not been previously described in *CHD7* disorder, in contrast with conotruncal defects being the most common heart defects, followed by atrioventricular septal defects [AVSD], arch vessel anomalies, and patent ductus arteriosus [PDA]. Of these, AVSD, conotruncal defects, and PDA are over-represented in patients with *CHD7* mutations compared to patients with nonsyndromic heart defects [20]. We would like to draw attention to the slight asymmetry of the eyelids at a young age as one patient presented with slight ptosis and another with a unilateral smaller eye slit, which is not considered a symptom of *CHD7* yet. 

Endocrine dysfunctions are frequently observed in individuals with CHARGE syndrome [21]. Roughly 60–80% of patients with CHARGE syndrome experience hypogonadotropic hypogonadism, characterized by genital hypoplasia and delayed or absent puberty due to gonadotropin-releasing hormone deficiency in both sexes. Growth retardation affects 60–72% of individuals with CHARGE syndrome, becoming evident from early infancy despite adequate fetal growth. This impaired growth can stem from feeding difficulties associated with cleft lip or palate, cardiac anomalies, and deficiencies in growth hormone.

The first two cases did not exhibit signs of genital hypoplasia or growth retardation. However, due to their prepubertal status, the gonadotropic axis cannot be estimated yet. Our third case has normal length and weight with no history of endocrine dysfunctions.

In search for genotype–phenotype correlations, the typical phenotype of CHARGE syndrome is often caused by nonsense and frameshift variants resulting in protein truncation and nonsense-mediated decay [14]. The *CHD7* variants identified in patients with an atypical presentation were predominantly missense variants [7]. Consequently, haploinsufficiency is hypothesized to be the primary pathogenic mechanism underlying CHARGE syndrome, whereas a defective but present protein might be associated with a less severe phenotype. 

The variant detected in case 1 has not been reported before to our knowledge, but three variants in the same intron 37 have. The c.8077-10T>A variant has been reported in a patient with atrioventricular septal defect, aortic coarctation, chorioretinal atrophy, bilateral progressive sensorineural hearing loss, and left kidney agenesis [22]. The variant induces an alternative splice site in intron 37, introducing eight bases in exon 38, resulting in a frameshift with premature stop codon and a prematurely truncated protein lacking 304 amino acids. The c.8077-2A>G was reported in a patient with electrolyte disorders, urogenital abnormalities, respiratory tract malformation, cardiovascular malformation, nervous system malformation, external ear malformation, and digestive system abnormality [23]. These findings might support the existence of an unknown functional domain in the last exon.

A c.6193C>A missense variant similar to those found in cases 2 and 3 has been reported before in a patient with semicircular canal aplasia, rhombencephalic dysfunction, hypothalamohypophyseal dysfunction, and mental retardation [24]. 

The *MYO3A* c.3133G>T p.(Val1045Leu) variant in cases 2 and 3 is not present in the gnomAD control population. This sequence alteration substitutes valine, which is neutral and non-polar, with leucine, also neutral and non-polar, at codon 1045 of the *MYO3A* protein (p.Val1045Leu). This variant has not been documented in the literature among individuals affected by *MYO3A*-related conditions. Theoretical prediction programs forecast a non-pathogenic effect. In summary, the available evidence is presently insufficient to determine the role of this variant in disease. Therefore, it has been classified as a Variant of Uncertain Significance.

The precise role of *CHD7* in the development and pathogenesis of audiovestibular disorders remains under investigation. Through its chromatin remodeling activity, *CHD7* appears to exert transcriptional control over hundreds of tissue-specific downstream genes [25,26]. SOX2 emerges as the top differentially expressed gene following *CHD7* deletion and is known to activate the hair cell initiator gene ATOH1. The *CHD7* S834F mutation has been shown to completely abolish *CHD7*’s ATPase and chromatin remodeling activities, resulting in the complete elimination of SOX2 expression. However, mono-allelic *CHD7* mutations resulted in only reduced SOX2 expression. Its absence in mouse otic tissues leads to the failure of hair cells and supporting cell generation [27,28]. Additionally, reduced SOX2 expression contributes to truncated semicircular canals, shortened cochleae, and hearing impairment [27]. SOX2 is only one of more genes of which the expression is controlled by *CHD7*. Another example is SOX9, which plays a crucial role in the early development of male reproductive organs by regulating the production of anti-Müllerian hormone [29,30,31]. Additionally, it is instrumental in cardiovascular organogenesis, proper craniofacial and tracheal development, with *CHD7* regulating neural crest cell development to promote normal morphogenesis [10,32].

Nie et al. [33] demonstrated its critical role in otic lineage specification and hair cell formation by creating an organoid with *CHD7* mutant human embryonic stem cell lines, including a complete knockout and a patient-specific missense mutant. The former resulted in the dysregulation of early otic lineage genes and a partially altered otic lineage identity, culminating in a total absence of both hair cells, and supporting cells. Conversely, a heterozygous *CHD7* deficiency resulted in the development of morphologically normal hair cells [34]. 

The above cases demonstrate the diversity in phenotype among *CHD7* variants, in which major or minor criteria of the CHARGE syndrome can also occur separately. They also highlight the variability of clinical and radiological findings, even within the same family and among individuals with the same mutation. It is crucial to consider this variability during genetic counseling for individuals with a known *CHD7* mutation who wish to have children. With 90–94% of individuals diagnosed with CHARGE syndrome exhibiting cochleovestibular dysfunctions and/or malformations [13], and *CHD7* mutations being responsible for the majority of cases, a multigene panel including *CHD7* appears justifiable in patients presenting with unilateral hearing loss or vestibular dysfunction and typical anomalies on MRI. Nevertheless, it remains appropriate to perform a complete screening for other associated abnormalities that fit within the CHARGE phenotype in patients with hearing loss and/or balance problems and a [likely] pathogenic *CHD7* variant.

## 5. Conclusions

The reported cases illustrate the phenotypical diversity associated with *CHD7* variants, of which some present with isolated cochleovestibular impairment. Consequently, *CHD7* should also be considered in nonsyndromic hearing loss, especially if there are ear anomalies and/or typical inner ear malformations on MRI. The importance of genetic counseling in view of a complete clinical work-up and discussion of inheritance implications is mandatory. This comprehensive strategy ensures a thorough understanding and management of potential health concerns related to *CHD7* variants.

## Figures and Tables

**Table 1:** CHARGE diagnostic criteria according to Verloes [3].

Verloes’ Diagnostic Criteria (2005)
Major Criteria Coloboma (iris or choroid, with or without microphthalmia) Choanal atresia Hypoplastic semi-circular Canals
Minor Criteria Rhombencephalic dysfunction (brainstem dysfunctions, cranial nerve VII to XII Palsies, and neurosensory deafness) Hypothalamo–hypophyseal dysfunction (including GH and gonadotrophin deficiencies) Abnormal middle or external ear Malformation of mediastinal organs (heart, esophagus) Mental retardation
Diagnostic Criteria Interpretation Typical CHARGE: 3 major or 2 major and 2 minor criteria Partial/incomplete CHARGE: 2 major and 1 minor criteria Atypical CHARGE: 2 major or 1 major and 3 minor criteria

## Data Availability

No new data were created or analyzed in this study. Data sharing is not applicable to this article.
